# Determination of Constraint-Independent Crack Tip Opening Angle for Stable Crack Growth in High-Strength Ductile Steels

**DOI:** 10.3390/ma18051051

**Published:** 2025-02-27

**Authors:** Xian-Kui Zhu

**Affiliations:** Materials Technology and Energy Science, Savannah River National Laboratory, Aiken, SC 29808, USA; xiankui.zhu@srnl.doe.gov

**Keywords:** fracture toughness, CTOA, constraint effect, DWTT, SENB, crack propagation

## Abstract

The crack tip opening angle (CTOA) is one of fracture toughness parameters that has been used for decades in describing large stable crack growth in thin-walled aerospace structures under the low-constraint conditions. Recently, the pipeline industry has developed a growing interest in using the CTOA parameter to serve as the minimum required fracture toughness to arrest dynamic crack propagation in modern gas transmission pipelines made of high-strength ductile steel. To meet this industrial need, the CTOA test standard ASTM E3039 was therefore developed for measuring the constant critical CTOA. ASTM E3039 recommends a drop weight tearing test (DWTT) specimen with a shallow crack for standard CTOA testing, but its CTOA may depend on the low constraint condition of the DWTT specimen at the crack tip. Verifying the constraint independence of the DWTT-measured CTOA thus becomes indispensable for applying CTOA toughness to the running fracture control in the pipeline design. For this purpose, the present paper evaluates critical CTOA values in a set of fracture toughness tests on single-edge notched bend (SENB) specimens with shallow and deep cracks, based on four CTOA estimation models. Among these, the Ln(P)-LLD linear fit model is similar to that recommended by ASTM E3039 for CTOA calculation. Fracture test data for X80 pipeline steel and HY80 structural steel were considered in the CTOA evaluation. The results showed that the four CTOA models were able to determine a constraint independent CTOA value for stable crack growth in the SENB specimens. As a result, a single, reliable, constant CTOA value could be determined regardless of the specimen geometry or the crack-tip constraint conditions. Therefore, the CTOA measured using ASTM E3039 is constraint-independent and transferable to use in cases of actual cracks propagating in gas transmission pipelines.

## 1. Introduction

Fracture mechanics methods play an indispensable role in structural engineering design and asset integrity management for national large-scale infrastructure, including pressure vessels and transmission pipelines. These pressure components are commonly made of either stainless steel or carbon steel, and both of these types of steel have good ductility against crack initiation or growth. For these ductile steels, elastic–plastic fracture mechanics methods are often utilized in engineering critical analysis (ECA), with the fracture toughness of the material characterized by one of the fracture parameters: J-integral [1], crack tip opening displacement (CTOD) [2], or crack tip opening angle (CTOA) [3]. The J-integral was originally proposed to describe the intensity of singularity of the crack tip field for a ductile material, CTOD was proposed to describe the capability of ductile crack openings, and CTOA was introduced to simulate stable crack growth in a ductile material for finite element analysis (FEA). Since the 1980s, these fracture parameters have also been used to describe the fracture toughness of ductile materials against crack initiation or growth. Over the past decades, many methods for measuring fracture toughness have been developed and standardized worldwide by different organizations, such as the American Society for Testing and Materials (ASTM) for metallic materials, as comprehensively reviewed by Zhu and Joyce [4]. Two commonly used standard fracture specimens for toughness testing are compact tension (CT) and single-edge notched bend (SENB) specimens. ASTM E399 [5] was the first standard to be developed for testing the plane strain fracture toughness K_IC_, and ASTM E1820 [6] was then developed for testing plane strain initiation toughness or resistance curves in terms of the J-integral or CTOD, where CTOD was converted from the J-integral. For non-standard fracture specimens, Zhu [7] presented a technical review of fracture toughness test methods under low-constraint conditions.

The J-integral and CTOD parameters are typically utilized to describe crack initiation and small stable crack growth [4], and the CTOD parameter is often employed to describe fracture toughness for pipeline girth welds [8,9]. In contrast, the CTOA parameter is particularly employed to describe fracture resistance against large stable crack growth in ductile steels [4]. The CTOA parameter has been used for decades as a reliable fracture toughness parameter to characterize large stable crack growth in thin-walled aerospace structures under low-constraint conditions [10]. ASTM developed the first CTOA test standard E2472 [11] in 2006 for thin-walled CT and middle-crack tension (MT) specimens, where CTOA was directly measured on the specimen surfaces using surface measurement technology such as digital image correction [12] or optical measurement methods [13]. In recent years, the pipeline industry started to use constant CTOA as the arrest fracture toughness parameter to control and prevent dynamic crack propagation in modern gas transmission pipelines [13,14,15,16,17,18,19,20,21]. The wall thickness in a gas pipeline typically ranges from 6.5 mm to 20 mm, which is considerably larger than the wall thickness (e.g., 5 mm or less) for aerospace structures. As a result, a surface CTOA measured using ASTM E2472 [11] may be not applicable to gas pipelines due to the thickness of the walls. Experiments have also shown that the CTOA measured at the midplane of a thicker fracture specimen can be significantly smaller than the measured surface CTOA of a thicker fracture specimen [21,22]. Thus, a constant midplane CTOA would be more appropriate for use in gas transmission pipelines. For this purpose, Martinelli and Venzi [23] developed an approximate model for estimating CTOA from the post-peak absorbed energy, which was calculated from load–displacement data measured from a single SENB specimen test. After modification, Xu et al. [24] proposed a simplified single-specimen method able to infer a more accurate CTOA value using the load–displacement data from a thicker drop weight tear test (DWTT) specimen. On this basis, ASTM developed a second CTOA test standard E3039 [25] in 2016, for measuring constant CTOA at the midplane in DWTT specimens for ferritic steels. The recommended DWTT specimens had a shallow crack, corresponding to low-constraint conditions at the crack tip. Without further study, it is unknown whether the ASTM E3039-measured CTOA is constraint-dependent or constraint-independent.

Constraint-dependent fracture toughness is often known as the constraint effect. The transferability issue of fracture toughness refers to whether the laboratory-measured fracture toughness is directly applicable to an actual crack without a constraint correction. The transferability of fracture toughness holds true when the laboratory-measured fracture toughness is constraint-independent. Otherwise, the application of a constraint correction on the measured fracture toughness must be considered to ensure that the constraint condition at an actual crack tip matches that of the laboratory fracture specimen. In order to prove the transferability of CTOA measured by the ASTM E3039 standard, many researchers [26,27,28,29,30,31,32] have studied the constraint dependence of CTOA measured from DWTT specimens or full-scale tests on different ductile steels, using experimental and numerical methods.

Parmar et al. [26] performed two FEA simulations on shallowly cracked DWTT specimens in three-point bending and in remote tension, respectively. The FEA results showed that the DWTT specimen in bending had a much higher constraint level at the crack tip than the DWTT specimen in tension. However, the critical CTOA values from these two loading conditions were comparable to the ASTM E3039 DWTT measured CTOA of 12.4° for X70 pipeline steel. It was concluded that “the crack-tip constraint condition has a negligible effect on the critical CTOA for the X70 pipeline steel”. In other words, the DWTT-measured CTOA at the midplane was constraint-independent. Recently, Zhen et al. [27] carried out a full-scale test (FST) on STPG370 carbon steel and performed a corresponding FEA simulation of CTOA for the tested burst pipe. The results demonstrated that the critical CTOA obtained from the FEA simulation was verified by the FST experimental data.

Recently, Jiao et al. [28] analyzed the dependency of the critical CTOA on the fracture speed obtained from different DWTT specimens for X80, with the CTOA decreasing as the fracture speed increased in the transition region of the DWTT specimens. Note that the CTOA values of the DWTT specimens were measured using high-speed cameras. Further study by Xu et al. [29] showed that (1) “the critical CTOA is insensitive to the fracture speed for the pipeline steel in the steady-state region of DWTT specimens that is relevant to the ductile fracture propagation in the gas pipeline”, and (2) “the ASTM E3039 determined CTOA is lose to the measured CTOA at the midplane of DWTT specimen”. In addition, Paermentier et al. [30] designed a dynamic tensile tear test (DT3) specimen that resembled the actual loading conditions of an in-service pipeline and conducted dynamic crack propagation tests to measure the critical CTOA for X70 pipeline steel. Their test results showed that the measured CTOA during the steady-state crack propagation in the DT3 specimens was constant at about 8° for the X70 pipeline steel.

To further study CTOA transferability, Shibanuma et al. [31] carried out a series of impact tests on DWTT specimens and a set of FSTs on an X70 line pipe. A high-speed camera was used to monitor the CTOA in pipes in both DWTT and full-scale burst tests. The experimental results showed that the CTOA remained constant during dynamic crack propagation in an X70 pipe at fracture speeds between 80 m/s and 200 m/s. After applying a correction for considering the tilt angle effect on the surface CTOA, Xu et al. [32] reported that the average surface CTOA values were similar to each other in both the DWTT (i.e., 20.1°) and FST (i.e., 20.9°). This indicates that the DWTT-measured CTOA is transferable to that for a running crack in an X70 pipeline.

Recently, Sun et al. [33] performed a dynamic fracture simulation of a running crack in a buried gas transmission pipeline using FEA models to consider the effects of soil constraints and gas decompression on the fracture speed and CTOA. The numerical results showed that the fracture speed decreased with decreasing crack tip pressure and the CTOA decreased with the increasing soil spring stiffness. Moreover, Bassindale et al. [20] and Zhen et al. [34] improved the Battelle two-curve model (BTCM) by using constant CTOA toughness to predict running fracture arrest in high-grade gas pipelines.

This paper aims to evaluate the constraint independence of CTOA in a different way from those discussed above. The CTOA was determined from quasi-static fracture toughness tests on SENB specimens with shallow and deep cracks in different constraint conditions at the crack tip. Four CTOA estimation models were used in the SENB testing to determine the constant CTOA associated with stable crack growth. Among these, the ln(P)-LLD linear fit model is similar to that used by ASTM E3039 for calculating CTOA from DWTT specimens. A series of fracture test data for SENB specimens with different crack sizes in high-strength ductile X80 and HY80 steels were considered in the CTOA evaluation. The results showed that the proposed CTOA models were able to determine crack size or the constraint-independent constant critical CTOA value associated with stable crack growth in the SENB specimens. This implies that the CTOA measured by ASTM E3039 is constraint-independent and transferable to use in the assessment of actual crack propagation in gas transmission pipelines.

## 2. CTOA Standard Test Methods

### 2.1. CTOA Definition

**Figure 1:**
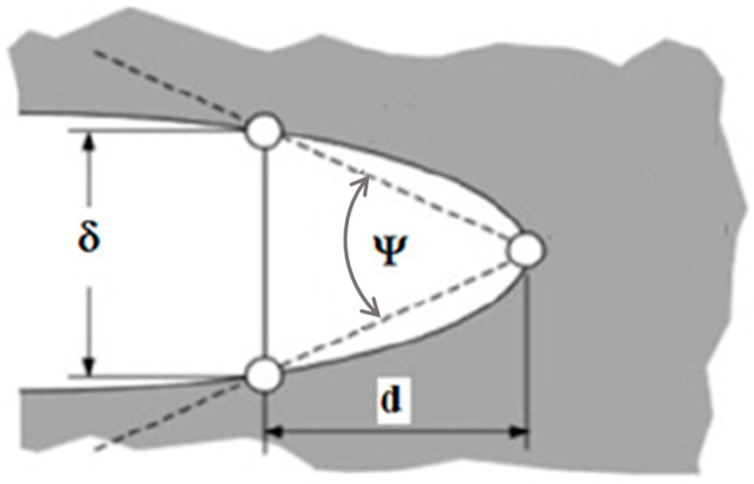
Illustration of CTOA at a blunt crack tip.

In fracture mechanics methods, the CTOA is defined as the angle between two faces of a crack starting from the crack tip. Due to significant blunting of sharp cracks in ductile steel, the original straight crack faces become curved during plastic deformation. For convenience, the CTOA is simply denoted by the angle ψ and the corresponding total CTOD is denoted by the symbol at a given distance *d* from the crack tip, as shown in Figure 1. The CTOA is mathematically expressed as follows:

(1) $$\psi =2\arctan\left(\frac{\delta }{2d}\right)$$

where the distance parameter d is typically given as a small value in the order of 1 mm. If the CTOA value is less than 20°, Equation (1) can be approximated as ψ = Δ/d with an error less than 1%.

### 2.2. CTOA Standard Test Method—ASTM E2472

In 2006, ASTM published the first standard method for testing CTOA in metallic materials in thin-walled specimens under low-constraint conditions, with the designation E2472 [11]. This CTOA standard is a direct surface measurement method and determines a constant critical CTOA associated with stable crack growth using a CT or MT specimen with anti-buckling guides. ASTM E2472 [11] determines an average CTOA within the specified crack extensions via Equation (1), using a four-point method.

To provide a longer uncracked ligament for larger stable crack growth, different thin-walled bending specimens such as modified double cantilever beams (MDCBs) [13] and DWTTs [35] have been employed to measure CTOAs in pipeline steels according to ASTM E2472 guidance [11]. Test results showed that two specimens had comparable CTOA values. In the context of CTOA testing, Xu et al. [35] pointed out that DWTT specimens were suitable for mill testing and MDCB specimens were more suitable for laboratory testing.

### 2.3. CTOA Standard Test Method—ASTM E3039

In 2016, ASTM developed a second CTOA standard test method, with the designation E3039 [25], for testing fracture propagation toughness in terms of constant CTOA using DWTT specimens. This standard method is applicable to ferritic steels exhibiting ductile fractures with 85% or more shear area. This CTOA test standard may meet the technical needs for improving CVN-based fracture control technology for managing modern gas transmission pipelines. The critical CTOA is defined at the midplane (B/2) of the DWTT specimen and calculated as follows:

(2) $$CTOA_{\frac{B}{2}}=\frac{8r_{p}}{\xi }\frac{180}{\pi }\;\left(degree\right)$$

where r_p_ is a rotation factor with an approximate constant of 0.55 and ξ is the absolute value of the slope of ln(P/P_m_) vs. the (Δ − Δ_m_)/S curve with specified data corresponding to ln(P/P_m_) values between −0.5 to −1.2, where P_m_ is the maximum applied force and Δ_m_ is the load-line displacement (LLD) at P_m_. The steady-state region of crack growth is assumed to occur between *P/P_m_* = 0.60 and *P/P_m_* = 0.30.

Numerical analysis [36] showed that the CTOA values obtained by the FEA calculation using Equation (2) were in good agreement with the values of CTOA_B/2_ calculated at the midplane of the DWTT specimen in the experiment. The results showed that the midplane CTOA_B/2_ was smaller than the surface CTOAc determined by ASTM E2472 [11].

### 2.4. Constant CTOA Simulation

In addition to the CTOA test methods and experimental studies, extensive numerical simulations have also been performed to study the constant CTOAs for various ductile steels [22,36,37]. Numerical simulations can determine more accurate CTOA values associated with stable crack growth in different fracture specimens, including CT, SENB, MT, MDCB, and DWTT. Once the constant critical CTOA_c_ (ψ_c_) toughness and crack driving force in terms of CTOA are obtained for a given crack, the crack stability can be assessed using the CTOA fracture criterion: CTOA ≤ ψ_c_. The resent work aims to determine a single, constraint-independent, constant CTOA toughness value for high-strength ductile steels rather than the CTOA based crack driving force.

## 3. CTOA Estimation Models for SENB Specimens

SENB and DWTT samples are three-point bending specimens, but an SENB sample may include smaller specimen sizes than a DWTT. For the same shallow crack ratio, these two types of bending specimens may have similar mechanical behavior at the crack tip, such as constraint conditions and stable constant CTOA at the midplane. This section introduces four indirect estimation models to evaluate the midplane CTOA from fracture toughness testing on SENB specimens under plane strain conditions.

### 3.1. CTOA Estimation from Load–Displacement Data

**Figure 2:**
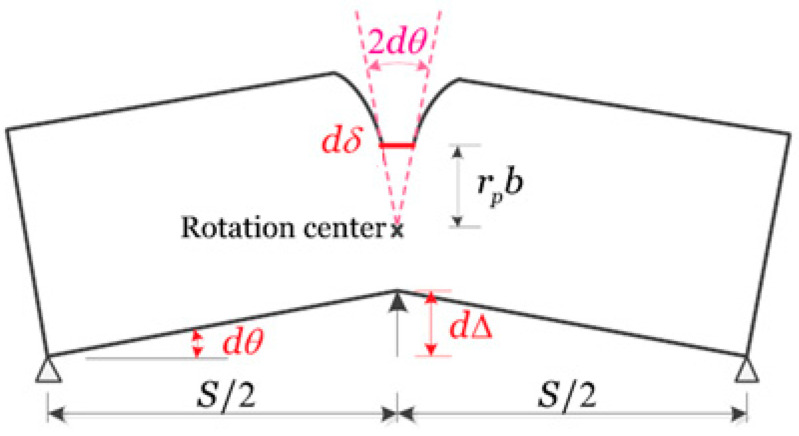
Plastic hinge model for an SENB specimen.

Recently, Zhu et al. [38] developed four CTOA estimation models for SENB specimens under plane strain conditions. Among these, the first three models were based on load–LLD data and the plastic hinge model, as recommended by BS 7448-1 [39], and the fourth model was based on the measured J-R curve. Figure 2 plots the plastic hinge model for an SENB specimen with a growing crack, where a small incremental LLD (dΔ) generates a small incremental CTOD (dδ) at the crack tip, a small incremental crack extension (d*a*), and a small rotation angle (dθ). The distance from the crack tip to the rotation center is denoted as r_p_b, with *b* = *W − a* denoting the ligament size and r_p_ the plastic rotation factor. For standard SENB specimens with a deep crack ratio within 0.45 ≤ a/W ≤ 0.70, the plastic rotation factor r_p_ = 0.44 [39]. For shallow-cracked SENB specimens, r_p_ may depend on crack size and the strain-hardening rate of the material [4]. This case is not discussed in this work.

A rigid plastic model requires the material to be perfectly plastic. For an SENB specimen in a perfectly plastic material, an applied load can be approximated as a limit load at the post, yielding the following:

(3) $$P=\frac{\lambda \sigma_{f}B{\left(W-a\right)}^{2}}{S}$$

where λ is a constant, σ_f_ is the flow stress defined as the average yield strength and ultimate tensile strength, *B* is the specimen thickness, *W* is the specimen width, *a* is the crack length, and *S* is the specimen span. For the limit load analysis of SENB specimens under plane strain conditions, the constant parameter λ = 1.455 [38]. For a strain-hardening material, however, λ may depend on the strain-hardening exponent of the material. In a single specimen test, the applied load and crack length are recorded during testing, and the constant parameter λ can thus be estimated from the test data and Equation (3).

From Figure 2, two simple geometrical relations are obtained as follows:

(4) $$d\theta =\frac{d\Delta }{S/2}$$

(5) $$d\delta =2r_{p}bd\theta$$

From the CTOA definition shown in Figure 1, when the distance d is replaced by da and δ is replaced by dδ, the resulting CTOA (ψ) in Equation (1) can be rewritten as follows:

(6) $$\tan\left(\frac{\psi }{2}\right)=\frac{d\delta }{2da}$$

From Equations (4)–(6), we can obtain the following relationship between CTOA, LLD, and crack extension:

(7) $$\tan\left(\frac{\psi }{2}\right)=\frac{2r_{p}b}{S}\frac{d\Delta }{da}$$

From Equation (3), we obtain the following differential load, dP:

(8) $$dP=-\frac{2\lambda \sigma_{f}Bb}{S}da$$

From Equations (7) and (8), after eliminating crack extension da, we obtain CTOA as a function of load and the slope of the load–LLD curve:

(9) $$\tan\left(\frac{\psi }{2}\right)=-\frac{4r_{p}P}{S}\frac{d\Delta }{dP}$$

When ψ ≤ 20^o^, tan(ψ/2) ≈ ψ/2 with an error less than 1%.

Assuming the crack grows stably from Point 1 (P_1_, Δ_1_, a_1_) to Point 2 (P_2_, Δ_2_, a_2_) on the P-LLD curve after the peak load and CTOA maintains a constant critical value (ψ_c_) during stable crack growth, in this situation, Equation (9) further derives three load–LLD-based CTOA models, as discussed below.

- **Model 1: P-LLD linear fit model**

For a linear portion of P-LLD data between Point 1 and Point 2, a linear curve fit can be expressed as follows:

(10) P=kΔ+c

where k and c are the linear curve fit constants. From Equations (9) and (10), the critical CTOA is obtained as follows:

(11) $$\tan\left(\frac{\psi_{c}}{2}\right)=-\frac{4r_{p}}{S}\left(\Delta +\frac{c}{k}\right)$$

- **Model 2: Ln(P)-LLD linear fit model**

Assuming the peak load point (P_max_, Δ_max_) is located on the P-LLD curve, Equation (9) can be expressed in the following format:

(12) $$\tan\left(\frac{\psi_{c}}{2}\right)=-4r_{p}\frac{d\left(\Delta -\Delta_{max}\right)/S}{d\left(Ln\left(\frac{P}{Pmax}\right)\right)}$$

Using the linear regression to curve fit the linear portion of the logarithmic load–LLD data from Point 1 to Point 2, Equation (12) can be used to calculate ψ_c_. If the CTOA is small (i.e., ψ_c_ ≤ 20°), tan(ψ_c_/2) ≈ ψ_c_/2 and Equation (12) reduces to Equation (2), as recommended by ASTM E3039 for DWTT specimens.

- **Model 3: Ln(P)-LLD exponential fit model**

Equation (9) can be restated in the following functional form of ln(P)—LLD:

(13) $$\tan\left(\frac{\psi }{2}\right)=-\frac{4r_{p}}{S}\frac{d\Delta }{d\left(\ln\left(P\right)\right)}$$

where an exponential function between ln(P) and LLD is assumed as the best-fitted curve from Point 1 to Point 2 on the measured data curve. Originally, Zhu et al. [38] assumed a fourth-order polynomial function to fit curve of the Ln(P)-LLD data associated with stable crack growth, but the current work found that an exponential fit function $\mathrm{Ln}\left(P\right)=C_{1}\mathrm{Exp}\left(C_{2}*\mathrm{LLD}\right)$, with C_1_ and C_2_ being curve fit constants, determines a better stable constant CTOA, as demonstrated in Section 5. Note that all three load–LLD models in Equations (11)–(13) contain only the rotation factor r_p_, but not the λ parameter.

### 3.2. J-Differential Estimation Method—Model 4

In fracture toughness testing of ductile steels, the J-R curve is evaluated using the incremental J-integral equation as recommended in ASTM E1820 [6], where the applied load (i.e., force), LLD, and crack length data are measured for a growing crack in a single specimen. For a quasi-statical crack, the differential of the J-integral is expressed as follows [40]:

(14) $$dJ=\frac{\eta }{Bb}Pd\Delta -\gamma \frac{J}{b}da$$

where η and γ are two LLD-based geometrical factors as a function of a/W.

From Equation (14), dΔ/da can be determined. Then, from Equation (7), the following J-differential equation is obtained for estimating the CTOA from the J-R curve:

(15) $$\tan\left(\frac{\psi }{2}\right)=\frac{2r_{p}}{\eta \lambda \sigma_{f}}\left(\frac{dJ}{da}+\frac{\gamma }{b}J\right)$$

The above equation is Model 4 for CTOA estimation, as proposed by Zhu et al. [38]. Originally, a J-R curve associated with stable crack growth was assumed to curve fit using a third- or fourth-order polynomial function. The current work found that a power law curve fit determines a better constant CTOA, as demonstrated in Section 5.

For standard SENB specimens, the two geometrical factors are often taken as η = 2 and γ = 1. Based on the SENB fracture test data and J-R curves obtained by Lam et al. [41] for A285 carbon steel, Zhu et al. [38] determined a set of slightly constraint-dependent CTOA values from the four CTOA estimation models described above, using a predetermined λ value. After the effect of fully plastic deformation on the λ parameter is correctly considered, a more accurate λ value was obtained from the SENB fracture test data for the A285 SENB specimens, and a constraint-independent, constant critical CTOA was determined based on the four CTOA estimation models for A285 carbon steel during stable crack growth [42]. The current work applied these four CTOA estimation models to determine the critical CTOA values during stable crack growth in X80 and HY80 high-strength ductile steels.

Recently, Lu and Wang [43,44] proposed another CTOA estimation model based on the K-R curve for a growing crack in a large thin-walled CT or MT specimen. This critical CTOA estimation requires elastically predominated deformation conditions to be maintained around the growing crack tip and is applicable only to thin-walled aircraft fuselage structures rather than thicker gas pipelines.

### 3.3. Comparisons of Four CTOA Models

As discussed above, four CTOA models were proposed by Zhu et al. [38] for evaluating the critical CTOA associated with stable crack growth in SENB specimens for ductile steels. Among these CTOA models, the first three were based on load–displacement data that were specifically applicable to SENB specimens (possibly for DWTT specimens), including Model 1 or the P-LLD linear fit model, Model 2 or the Ln(P)-LLD linear fit model, and Model 3 or the Ln(P)-LLD exponential fit model. The results in Section 5 show that these three CTOA models predicted comparable results on average. Model 1 predicted linearly distributed CTOA values over the region of interest, Model 2 was found to be equivalent to the CTOA model recommended by ASTM E3039 for DWTT specimens and predicted a constant CTOA value over the region of interest, and Model 3 predicted nonlinearly distributed CTOA values over the region of interest. Model 4, namely the J-differential CTOA estimation model, is completely different from the first three CTOA models. This model provides a physical relationship between the J-integral and the CTOA and predicts the CTOA value from the corresponding J-R curve. Model 4 may be applicable to any fractured specimen provided the four geometry parameters of η, γ, λ, and r_p_ are available for that specimen.

## 4. Fracture Resistance Testing Using SENB Specimens

### 4.1. Fracture Resistance Testing for X80 Pipeline Steel

Six SENB specimens were tested by Shen et al. [45] at room temperature (i.e., 20 °C) to develop J-R curves for X80 pipeline steel according to ASTM E1820 guidance [6], with varying initial crack lengths to achieve different constraint levels at the crack tip under plane strain conditions. These SENB specimens were machined from a 48-inch X80 pipe. The chemical compositions of this material were reported by Shen et al. [45]. The uniaxial tensile test obtained a 0.2% offset yield stress of 570 MPa (82.7 ksi) and an ultimate tensile stress (UTS) of 675 MPa (97.9 ksi), leading to flow stress (σ_f_) of 622.5 MPa (90.3 ksi) and Y/T of 0.844. This indicates that X80 is a high strength carbon steel with a low strain-hardening rate.

All the SENB specimens had a width W = 23 mm, thickness B = W/2 = 11.5 mm, net thickness B_N_ = 9.2 mm due to a 10% side groove on each side, and the beam span S = 4W = 92 mm. These SENB specimens were pre-cracked by fatigue under applied three-point bending load conditions. After pre-cracking, the initial crack ratios a_o_/W of the six SENB specimens were measured as 0.24, 0.25, 0.42, 0.43, 0.63, and 0.64. These SENB specimens were thus categorized as two shallow-, two intermediate-, and two deep-cracked fracture specimens.

**Figure 3:**
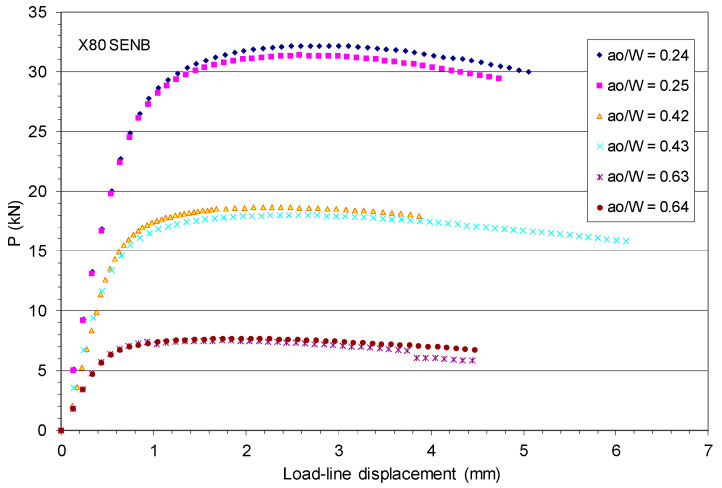
Experimental P–LLD data for six X80 SENB specimens.

Figure 3 shows the test load–LLD data recorded for the six X80 SENB specimens with a_o_/W = 0.24 to 0.64 [45].

As shown in Figure 3, all six SENB specimens initially experienced linearly elastic response, whereby the load linearly increased with the LLD. Then, plastic strain hardening occurred and the applied load increased nonlinearly with the plastic deformation up to the maximum load and then dropped until the testing was terminated or the specimen failed. The maximum load of these SENB specimens decreased dramatically as the initial crack length increased. For example, the maximum load P_max_ = 32.2 kN, 18.6 kN, and 7.5 kN, respectively, for a_o_/W = 0.24, 0.42, and 0.63.

**Figure 4:**
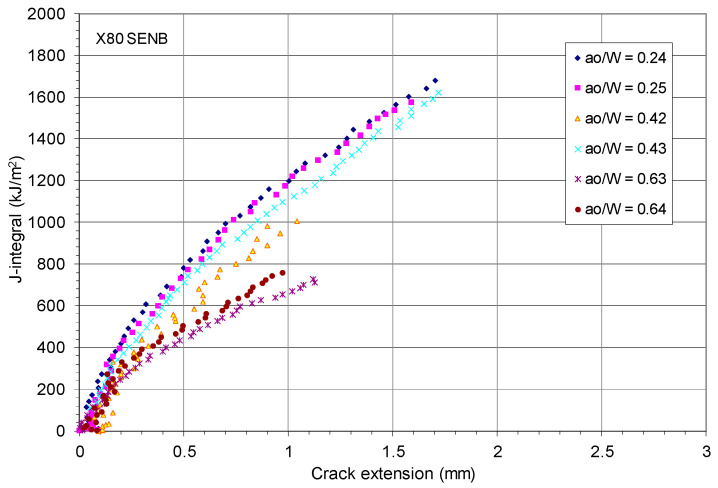
Experimentally measured J-R curves for six X80 SENB specimens.

Figure 4 shows the experimentally measured J-R curves [45] that were developed by following the ASTM E1820 fracture test procedures based on the load, LLD, and crack length data obtained during the fracture test where the crack length was measured using the elastic unloading compliance method. Figure 4 clearly shows the dependence of the J-R curves on crack size and the constraint effect on the J-R curves in the X80 pipeline steel. The fracture toughness test results showed that the initiation fracture toughness J_Ic_ of the X80 steel ≈ 400 kJ/m^2^.

Initially, this work intended to use the measured P-LLD data and experimental data of J-R curves of the X80 SENB specimens to evaluate the CTOA models described above. However, Figure 4 shows that the crack extensions in all X80 SENB fracture tests were too short, and stable crack growth may not have started for these cracks, particularly the deeper ones. As a result, these X80 SENB test data are not adequate to use for an evaluation of constant critical CTOA.

Recently, Zhen et al. [46] performed a series of FEA simulations of crack propagation in X80 pipeline steel using SENB, CT, and MDCB specimens with a wide range of initial crack lengths. The yield strength of this X80 steel was 582 MPa (84.4 ksi) and the UTS was 696 MPa (100.9 ksi). These material properties were similar to those of the X80 pipeline steel used by Shen et al. [45], and the fracture toughness properties of these two X80 pipeline steels should thus be comparable. The numerical simulations aimed to determine the constraint effect on the critical CTOA during stable crack tearing due to different specimen sizes, crack sizes, and specimen configurations. In order to simulate crack propagation, the authors employed ABAQUS/Explicit solver [47] and the GTN micromechanical damage model developed by Gurson [48] and Tevergaard and Needleman [49] for all their elastic–plastic FEA simulations under both 2D plane strain conditions and 3D conditions.

**Figure 5:**
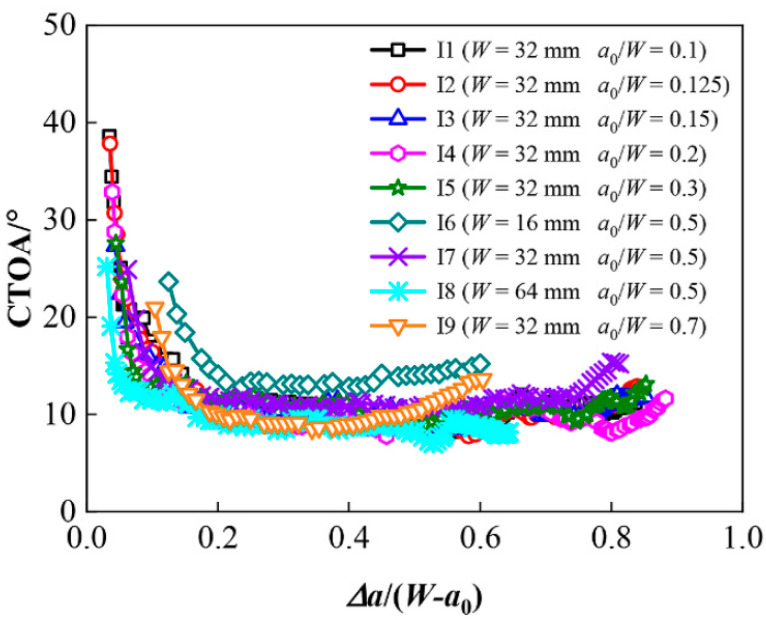
Numerical results of CTOA during crack growth determined from X80 SENB specimens with different crack sizes.

Figure 5 shows the FEA numerical results of the CTOD values during crack growth obtained by Zhen et al. [46] for X80 SENB specimens under 2D plane strain conditions, where three specimen widths of W = 16 mm, 32 mm, and 64 mm and seven initial crack depth ratios of a_o_/W = 0.1, 0.125, 0.15, 0.2, 0.3, 0.5, and 0.7 were employed in FEA simulations of crack propagation. Figure 5 shows that (1) all the CTOA-resistance curves initially had a high value and then experienced sudden drop during early extension of the cracking; (2) all cracks grew into the steady-state extension at Δa/(W − a_o_) ≈ 0.2; (3) the constant steady-state value of CTOA was on average ~10° degrees within the crack growth region of interest; (4) the thinnest specimen (W = 16 mm) with a_o_/W = 0.5 had a slightly larger CTOA value; and (5) all the deep cracks seemed to have an upward CTOA trend at the end of the stable crack growth. From these observations, Zhen et al. [46] concluded that “the arrest toughness CTOA_C_ for X80 pipeline steel is not sensitive to the change of in-plane constraint levels at the crack tip for SENB and CT specimens”.

Regarding the starting point of the stable crack tearing, Δa/(W − a_o_) ≈ 0.2, as shown in Figure 5, this represents the start of stable ductile crack growth at Δa ≈ 1.6 mm for W = 16 mm and a_o_/W = 0.5, Δa ≈ 3.2 mm for W = 32 mm and a_o_/W = 0.5, and Δa ≥ 4.48 mm for all other crack sizes in the X80 SENB specimens. Comparison of these starting points of stable ductile crack growth with the maximum crack extensions measured by Shen et al. [45], as shown in Figure 4, confirms that the fracture test data obtained from the X80 SENB specimens, including the measured load–LLD data shown in Figure 3 and the experimental J-R curves shown in Figure 4, are not sufficient to quantify the corresponding constant critical CTOA value due to absence of stable crack growth zone.

### 4.2. Fracture Resistance Testing of HY80

As an alternate to the X80 pipeline steel used by Shen et al. [45], an HY80 structural steel [50] is considered here because these two steels are high-strength ductile steels with the same minimum nominal yield stress of 80 ksi (552 MPa). While X80 is a high-strength carbon steel for pipelines, HY80 is a high yield (HY)-strength submarine steel with low carbon and low alloy and has been used for shipbuilding for more than 55 years [51].

A series of fracture toughness test results for HY80 steel was reported by Joyce and Link [50] in 1997. All of these fracture tests were conducted on HY80 SENB specimens at room temperature (21 °C) according to ASTM E1820 guidance [6], and the initial crack lengths varied from shallow to deep in order to develop different constraint levels at the crack tip. The SENB specimens were machined from 27 mm thick plate in HY80 steel. Chemical compositions of this material are provided in Ref. [50]. Uniaxial tensile testing determined a 0.2% offset yield stress of 630 MPa (91.4 ksi) and a UTS of 735 MPa (106.6 ksi), leading to a flow stress (σ_f_) of 682.5 MPa (99.0 ksi) and Y/T = 0.857. This high Y/T ratio indicates that HY80 is a low-strain hardening steel, similar to X80.

All the HY80 SENB specimens had a 1T standard specimen width W = 50.8 mm (2 in.), specimen thickness B = W/2 = 25.4 mm (1 in.), net thickness B_N_ = 20.32 mm (0.8 in.) due to 20% side grooves, and beam span S = 4W = 203 mm (8 in.). These SENB specimens were pre-cracked by fatigue via three-point bending. After pre-cracking, the initial crack ratios a_o_/W of the SENB specimens were measured to vary in the range of 0.135 to 0.83. In general, deep cracks in the SENB specimens generate high constraint levels at the crack tip, and shallow cracks in the SENB specimens generate low constraint levels at the crack tip.

**Figure 6:**
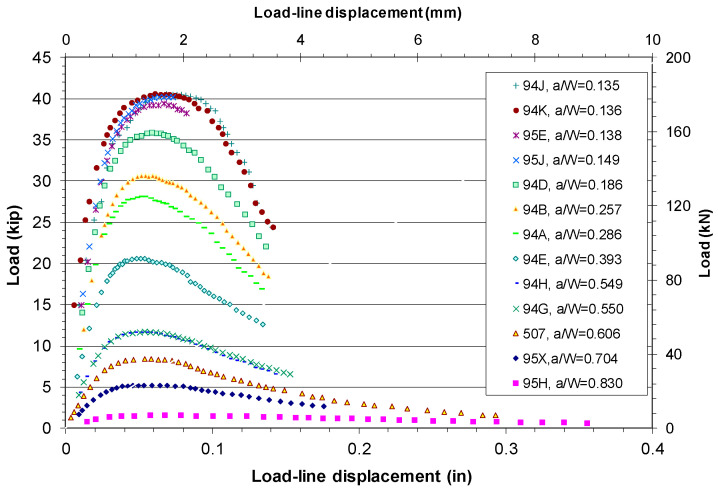
Experimental P–LLD data for a series of HY80 SENB specimens.

Figure 6 shows the load–LLD test data measured during the fracture toughness testing on thirteen HY80 SENB specimens with a_o_/W = 0.135 to 0.83 [50].

As shown in Figure 6, the applied load initially increased with LLD in all the HY80 SENB specimens. Then, plastic strain hardening occurred, and the applied load increased nonlinearly with LLD up to the maximum load and then dropped until the specimen failed. The maximum load of these SENB specimens decreased dramatically as the initial crack length increased. For example, the maximum load P_max_ = 180.2 kN, 91.8 kN, and 37.3 kN, respectively for a_o_/W = 0.136, 0.393, and 0.606.

In the original fracture toughness testing using HY80 SENB specimens, the elastic unloading compliance method recommended by ASTM E1820 [6] was adopted for monitoring crack length and for determining crack growth. Experimentally measured J-R curves were reported by Joyce and Link [50] for HY80 SENB specimens. It should be noted that their experimental data of J-R curves for shallow-cracked SENB specimens with a_o_/W ≤ 0.282 may be incorrect because a negative γ factor was used for the shallow cracks. In the J-R curve evaluation described by ASTM E1820 [6], the η factor equation is used for calculating the incremental deformation J-integral at the current load step, and the γ factor is used for correction of crack growth on the increment of the J-integral. This implies that the γ factor must be positive because a negative γ factor increases the deformation J-integral, which has no physical meaning (see Zhu and Joyce [52] for further discussion). For this reason, the original J-R curves developed by Joyce and Link [50] are not reported here.

**Figure 7:**
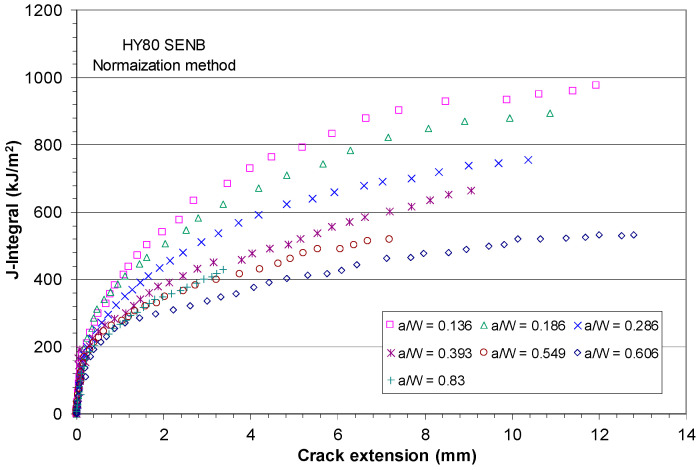
Experimental J-R curves for a set of HY80 SENB specimens.

Ten years later, in 2007, Zhu and Joyce [52] revisited fracture toughness testing on HY80 SENB specimens and redetermined the crack lengths and J-R curves using the normalization method recommended in Annex 15 of ASTM E1820 [6]. Note that more accurate expression of the η factor was developed and a non-negative expression of the γ factor was obtained by Zhu and Joyce [52]. These new expressions of the η and γ factors were used for reevaluation of the J-R curves. Figure 7 shows the experimental J-R curves that were developed by Zhu and Joyce [52] following ASTM E1820 procedures and based on the applied load, LLD, and crack length obtained from the fracture tests, where the crack length was estimated using the normalization method.

Figure 7 clearly shows the strong size-dependence of the J-R curves of the HY80 steel specimens; the in-plane constraint level at the crack tip had a significant effect on the fracture resistance curves. The fracture test results showed that the initiation fracture toughness J_Ic_ ≈ 200 kJ/m^2^ in the standard SENB specimens of HY80 steel. Compared with the initial fracture toughness J_Ic_ ≈ 400 kJ/m^2^ for the X80 steel, this reveals that the HY80 submarine steel had significantly lower fracture toughness than the X80 pipeline steel, although these two steels had a similarly high yield strength of 80 ksi.

Recall that ASTM E1820 [6] requires the standard SENB specimens to have initial crack sizes within 0.45 ≤ a_o_/W ≤ 0.7. Figure 7 shows that all non-standard SENB specimens with a_o_/W < 0.45 or a_o_/W > 0.7 presented elevated J-R curves compared with the standard conservative J-R curves for HY80 SENB specimens with a_o_/W = 0.549 and 0.606.

As shown in Figure 7, all crack extensions for the HY80 SENB specimens were larger than 7 mm, which was much larger than those at the starting points of the stable ductile crack growth for X80, as shown in Figure 5. Therefore, these HY80 SENB fracture test data would be adequate for the evaluation of constant CTOA associated with longer stable crack growth in HY80 high strength ductile steel.

## 5. Determination of Constant CTOA for HY80

### 5.1. Determination of λ Parameter

**Figure 8:**
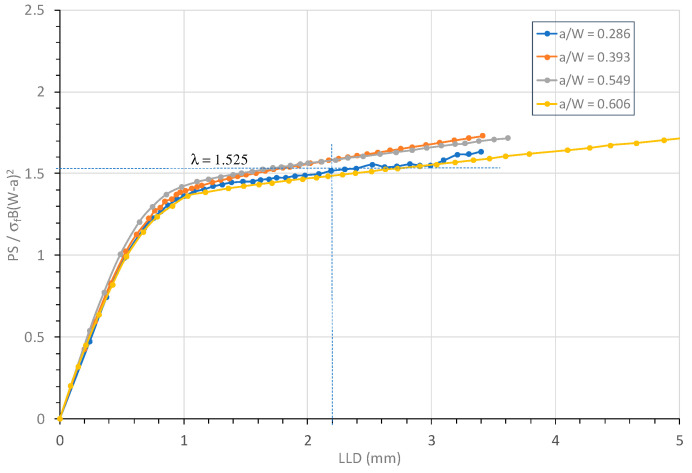
Variation of λ parameter with LLD for four HY80 SENB specimens.

The J-differential method requires the λ parameter value in Equation (15) in order to calculating the CTOA associated with stable crack extension. The λ parameter in Equation (3) is a predefined constant for a perfectly plastic material but may depend on the strain-hardening exponent of the material for a strain-hardening material. The λ parameter can be estimated from the applied load and crack size data measured during fracture testing. From the fracture test data, the λ parameter can be calculated for each specimen over the entire deformation, as shown in Figure 8.

It can be seen from Figure 8 that the calculated λ value during the plastic deformation varied from 1.25 to 1.75 and was nearly independent of crack size. Figure 6 shows that for all SENB tests, LLD ≈ 2.4 mm corresponded to a stable crack growth stage during which an approximate linear relation existed between load and LLD. At this LLD, λ = 1.525 was estimated according to Figure 8. This λ parameter value is used in the current work for the critical evaluation of the CTOA in HY80 steel.

**Figure 9:**
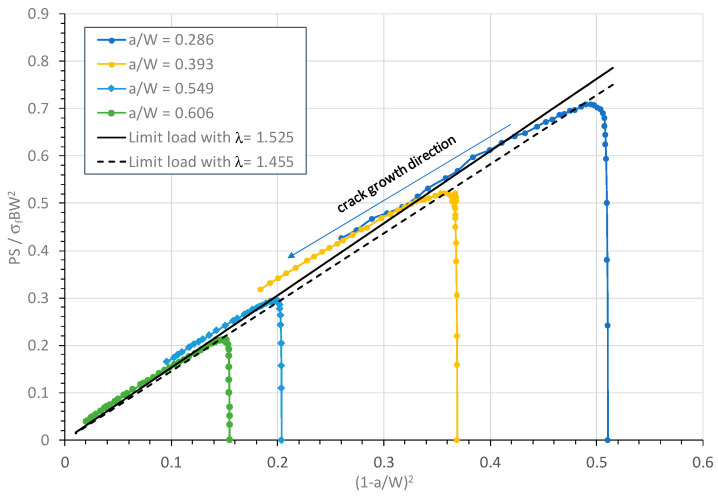
Comparison of limit load with applied load in fracture tests for four HY80 SENB specimens.

Figure 9 shows variations of the applied load P and the limit load solution from Equation (3) with the ligament-squared value (1 − a/W)^2^ during the entire deformation, including initial elastic deformation and plastic deformation during crack growth. In Figure 9, both the applied and limit loads have been normalized using σ_f_ BW^2^/S. This figure includes two limit load solutions, one using the estimated λ = 1.525 for HY80 steel and the other using λ = 1.455 for a perfectly plastic material. For each HY80 SENB specimen, the normalized applied load increased quickly from an initially small load in the elastic deformation condition to the maximum load in the full plastic deformation condition and then decreased gradually in a linear manner due to the stable crack growth. The direction of crack growth is marked in Figure 9. Clearly, the limit load solution with λ = 1.525 is a better match with the experimental data and better able to describe the fully plastic conditions of the HY80 SENB specimens compared with λ = 1.455 for the perfectly plastic material. Thus, λ = 1.525 was selected for use hereafter.

### 5.2. CTOA for HY80 SENB with a_o_/W = 0.606

This section determines the critical CTOA value for each HY80 SENB specimen in terms of Model 1 in Equation (11), Model 2 in Equation (12), Model 3 in Equation (13), and Model 4 in Equation (15). To quantify the constraint effect on the critical CTOD, four HY80 SENB tests were selected and are analyzed in this section. These included crack sizes of a_o_/W = 0.606, 0.549, 0.393, and 0.286 in the HY80 SENB specimens. These four crack sizes reflect different constraint effects on the J-R curves, as shown in Figure 7, and describe high to low levels of constraint at the crack tip in the HY80 SENB specimens.

It is commonly known that experimental test data always contain certain variations due to measurement noise, and a smoothed best-fit curve is thus needed for determining the first-order derivative of the best-fit curve. For Model 1, the linear curve fit was used to simply fit the experimental data exhibiting a linear relation on the measured P-LLD curve, and the CTOA was calculated from Equation (11). For Model 2, the peak load point (P_max_, Δ_max_) was first located, and Ln(P/P_max_) and (Δ − Δ_max_)/S were then calculated from the peak load point to the final measured point. Linear regression was used to fit the linear portion of the Ln(P/P_max_) vs. (Δ − Δ_max_)/S data, and the CTOA was calculated from Equation (12). For Model 3, Ln(P) was first calculated, and an exponential function was curve-fitted on the Ln(P)-LLD data. The first-order derivative of the exponential curve was calculated, and the CTOA was determined from Equation (13). For Model 4, a power law function was curve-fitted using the nonlinear regression method from the J-R curve obtained in the fracture test. Then, the first-order derivative of the power law J-R curve was calculated, and the CTOA was finally determined from Equation (15).

**Figure 10:**
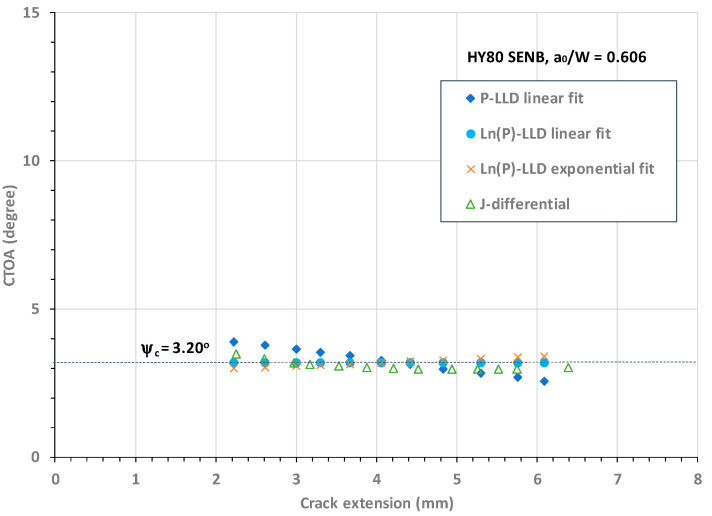
CTOA resistance against crack extension for HY80 SENB specimen with a_o_/W = 0.606.

Using these procedures, the CTOA resistance curves (or constant CTOA) against stable crack growth were determined using the three load–LLD-based models and the J-differential model. Figure 10 plots the CTOA resistance curves against crack extension, with the CTOA values obtained using Models 1, 2, 3, and 4, respectively, for an HY80 SENB specimen with a deep crack of a_o_/W = 0.606. It was found that all the proposed models determined comparable critical CTOA values over a range of stable crack extension from Δa = 2.0 mm to 6.2 mm. The following observations were made regarding the stable crack growth zone: (1) Model 2 determined a constant CTOA, ψ_c_ = 3.20°; (2) Model 1 determined a linearly decreasing CTOA with an average value of ψ_c_ = 3.18° with an R-square value of 0.9982; (3) Model 3 determined a nearly constant CTOA with an average value of ψ_c_ = 3.19° and an R-squared value of 0.9982; and (4) Model 4 was able to determine the CTOA associated with large crack growth, and the CTOA curve became nearly flat over the stable crack growth zone with an average value of ψ_c_ = 3.10° and an R-squared value of 0.9827. These high R-squared values indicate the high quality of the predefined curve-fit functions.

### 5.3. CTOA for HY80 SENB with a_o_/W = 0.549

**Figure 11:**
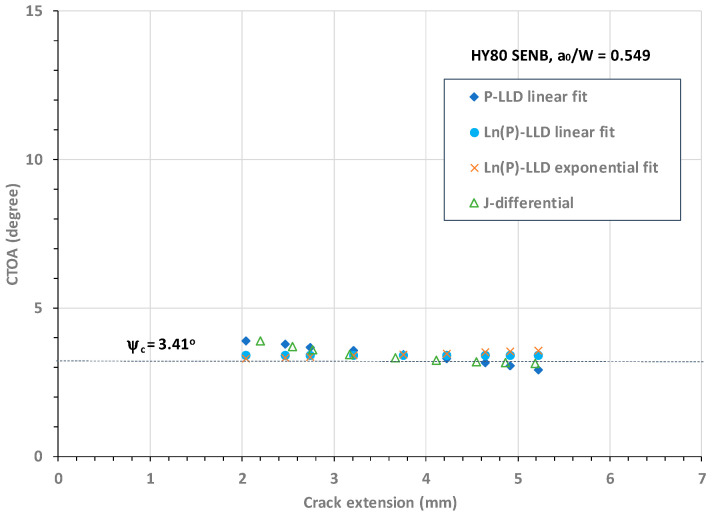
CTOA resistance against crack extension for HY80 SENB specimen with a_o_/W = 0.549.

In the same manner as discussed above, the four best curve-fitted functions were obtained and then utilized to calculate the CTOA. Figure 11 plots the CTOA resistance against crack extension obtained using the four CTOA models for an HY80 SENB specimen with a deep crack of a_o_/W = 0.549.

As evident in Figure 11, all four CTOA models determined comparable critical CTOA values over the range of stable crack extension from Δa = 2.0 mm to 5.0 mm. Figure 11 demonstrates that regarding the stable crack growth zone, (1) Model 2 determined a constant CTOA, ψ_c_ = 3.41°, (2) Model 1 determined a linearly decreasing CTOA with an average value of ψ_c_ = 3.42°, (3) Model 3 determined a nearly constant CTOA with an average value of ψ_c_ = 3.43°, and (4) Model 4 also determined a nearly constant CTOA over the stable crack extension zone with an average ψ_c_ = 3.40°.

### 5.4. CTOA for HY80 SENB with a_o_/W = 0.393

**Figure 12:**
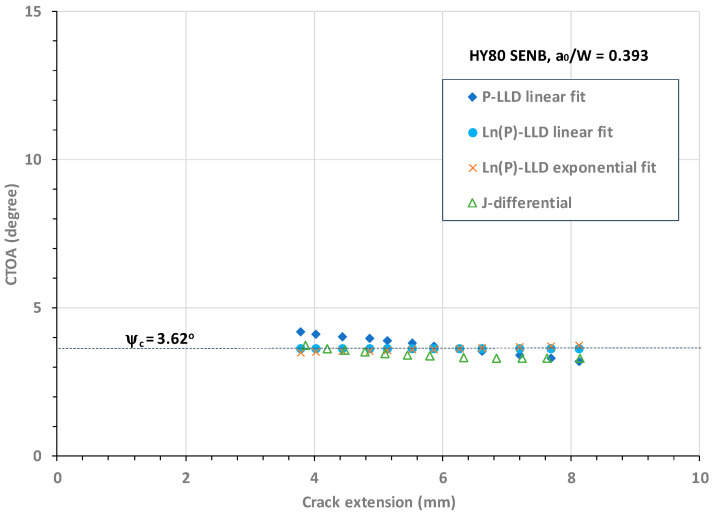
CTOA resistance against crack extension for HY80 SENB specimen with a_o_/W = 0.393.

In the same manner, the four best curve-fitted functions were obtained and employed to determine the CTOA values. Figure 12 plots the CTOA resistance against crack extension obtained from Models 1, 2, 3, and 4, respectively for an HY80 SENB specimen with an intermediate crack size of a_o_/W = 0.393.

Figure 12 shows that all four CTOA models determined comparable critical CTOA values over the stable crack extension from Δa = 3.8 mm to 8.1 mm.

In Figure 12, it can be observed that regarding the stable crack extension zone, (1) Model 2 determined a constant CTOA, ψ_c_ = 3.62°, (2) Model 1 determined a linearly decreasing CTOA with an average constant value of ψ_c_ = 3.63°, (3) Model 3 determined a nearly constant CTOA with an average value of ψ_c_ = 3.61°, (4) Model 4 also determined a nearly constant CTOA over the stable crack growth zone with an average constant value of ψ_c_ = 3.44°, and (5) Model 4 determined conservative CTOA values associated with most of the stable crack extensions. These lower CTOA values may have been caused by use of the constant η = 2 for all crack sizes in the HY80 SENB specimens, because this η value was used in the experimental J-R curve evaluation [52]. If a smaller η value (e.g., η = 1.9 as recommended in the current ASTM E1820) were used, the CTOA value would go up.

### 5.5. CTOA for HY80 SENB with a_o_/W = 0.286

**Figure 13:**
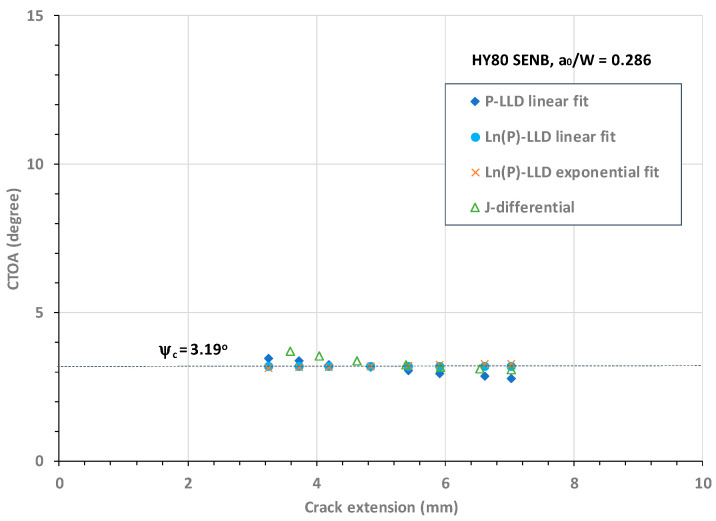
CTOA resistance against crack extension in the HY80 SENB specimen with a_o_/W = 0.286.

In a similar way, Figure 13 shows the CTOA resistance against crack extension obtained from four CTOA models for an HY80 SENB specimen with a shallow crack size of a_o_/W = 0.286.

Figure 13 shows that all four CTOA models determined comparable critical CTOA values over the stable crack extension from Δa = 3.2 mm to 7.0 mm. From Figure 13, it can be observed that regarding the stable crack growth zone, (1) Model 2 determined a constant CTOA, ψ_c_ = 3.19°, (2) Model 1 determined a linearly decreasing CTOA with an average constant value of ψ_c_ = 3.21°, (3) Model 3 determined an almost constant CTOA with an average value of ψ_c_ = 3.21°, and (4) Model 4 determined a slightly decreasing CTOA with an average constant value of ψ_c_ = 3.31°. Note that η = 2 was used in the original experimental J-R curve evaluation of this shallow crack. In fact, the η factor for this shallow crack is not a constant but may vary as the crack extends.

In summary, the above analyses as shown in Figure 10, Figure 11, Figure 12 and Figure 13 demonstrate that (1) the Ln(P)-LLD linear fit model (i.e., Model 2) determined a constant CTOA associated with stable crack growth for shallow and deep cracks in HY80 SENB specimens; (2) the P-LLD linear fit model (i.e., Model 1) determined a linearly decreasing CTOA associated with stable crack growth for all cracks; (3) the Ln(P)-LLD exponential fit model (i.e., Model 3) determined a nearly constant CTOA associated with stable crack growth for all cracks; and (4) the J-differential model (i.e., Model 4) determined a slightly conservative CTOA associated with stable crack growth. Accurate CTOA results depend on the quality of J-R curve test data and the exponential curve fit over the entire range of crack extension.

### 5.6. Constraint Independence of Critical CTOA

**Figure 14:**
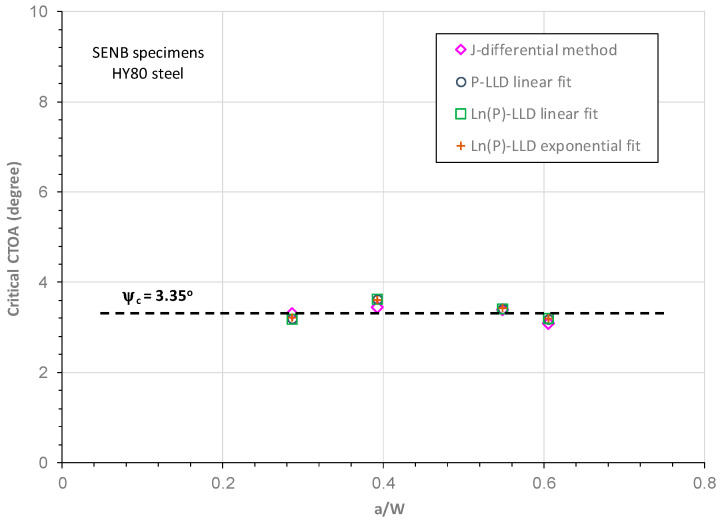
Variations of constant critical CTOA with crack sizes for an HY80 SENB specimen.

Figure 14 compares the critical CTOA values obtained in this work with the crack sizes in the HY80 SENB specimens. As shown in Figure 14, the crack size or crack-tip constraint level had a small or negligible effect on the critical CTOA value, and the average critical CTOA for the HYU80 steel was obtained as 3.35°. In contrast, the crack-tip constraint level had a significant effect on the experiemntal J-R curves of HY80 steel, as shown in Figure 7. J-R curves are frequently used to characterize fracture resistance against ductile crack initiation and short stable crack tearing in ductile steels, and the current results demonstrate that the constant CTOA toughness is an adequate fracture parameter to describe large stable crack growth in high-strength ductile steels.

## 6. Conclusions

This paper evaluates the constraint independence of the critical CTOA values that were determined in relation to stable crack growth using the fracture test data for SENB specimens and based on four CTOA estimation models. A series of experimental test data on SENB specimens of HY80 structural steel was employed to assess these CTOA models and to determine the constraint independence of the critical CTOA in HY80 steel. From the present study and other available numerical and experimental investigations on the transferability of CTOA measured using DWTT specimens, the primary results obtained are as follows:

(1) The Ln(P)-LLD linear fit model determined a constant critical CTOA associated with stable crack growth. The P-LLD linear fit model determined a linearly decreasing CTOA, and the Ln(P)-LLD exponential fit model determined a nearly constant CTOA associated with stable crack growth. On average, these load–displacement-based models determined comparable CTOA values associated with stable crack growth;
(2) Using experimental J-R curve data and a power law curve fit, the J-differential model determined a nearly constant critical CTOA value associated with stable crack growth. This CTOA value was comparable to those determined from the three-displacement based models. Note that the J-R curves for HY80 steel strongly depended on the constraint levels at the crack tip, whereas its critical CTOA value was essentially independent of the crack-tip constraint levels associated with stable crack growth. Therefore, J-R curves are usually used to characterize ductile crack initiation and small stable crack tearing in ductile materials, and the constant critical CTOA is the best fracture parameter to use for describing large stable crack growth for high-strength ductile steels;
(3) The results showed the Ln(P)-LLD linear fit model to be comparable to the CTOA model recommended by ASTM E3039 and determined a constant critical CTOA associated with the stable crack growth in all cracks in the SENB specimens. The other three CTOA models also determined comparable constant critical CTOAs associated with stable crack growth;
(4) X80 and HY80 are both high-strength steels with a similar yield strength of 80 ksi (552 MPa), but they have significantly different fracture toughness values. For X80, fracture toughness J_Ic_ = 400 kJ/m^2^ and the critical CTOA = 10°. For HY 80, fracture toughness J_Ic_ = 200 kJ/m^2^ and the critical CTOA = 3.35°;
(5) The results demonstrated that the constant critical CTOA determined using HY80 SENB specimens was independent of crack size or constraint level at the crack tip. This implies that the CTOA measured by ASTM E3039 is constraint-independent and thus supports the transferability of CTOA measurement by ASTM E3039 to actual crack assessment. Therefore, the CTOA is a single, reliable, constant measurement of fracture toughness regardless of fracture specimen geometry or constraint level at the crack tip. As a result, the CTOA fracture criterion allows accurate prediction of crack propagation or arrest in complex structures, which can improve design analysis and enhance robust characterization of materials.

## Data Availability

The original contributions presented in this study are included in the article. Further inquiries can be directed to the corresponding author.
